# Electrochemical investigation of the interaction of 2,4-D and double stranded DNA using pencil graphite electrodes

**DOI:** 10.3906/kim-2011-56

**Published:** 2021-06-30

**Authors:** Gülşah ÇONGUR

**Affiliations:** 1 Vocational School of Health Services, Bilecik Şeyh Edebali University, Bilecik Turkey; 2 Biotechnology Application and Research Center, Bilecik Şeyh Edebali University, Bilecik Turkey

**Keywords:** DNA-herbicide interaction, 2,4-dichlorophenoxyacetic acid, pencil graphite electrode, voltammetric analysis, electrochemical characterization, microscopic characterization

## Abstract

2,4-dichlorophenoxyacetic acid (2,4-D) is an auxinic herbicide used to control broadleaf weeds. It is also a threatening factor for not only aquatic life but also human health due to its genotoxicity and endocrine disruptive property. Herein, the interaction between 2,4-D and double stranded DNA was investigated by using single-use pencil graphite electrodes (PGE) in combination with electrochemical techniques. The detection mechanism was based on the monitoring of the changes at the guanine oxidation signal obtained before/after surface-confined interaction of 2,4-D and DNA at the surface of PGE. The electrochemical characterization of the interaction was studied by using microscopic and electrochemical techniques. The response obtained by interaction in the presence of another herbicide, glyphosate, which is widely used with 2,4-D for weed control, was compared to the one occurred in the presence of 2,4-D. Electrochemical monitoring of the interaction between the herbicide whose active molecule was 2,4-D and DNA was also investigated. The detection (LOD) and quantification limits (LOQ) for 2,4-D and the herbicide could be obtained in the linear concentration ranges of 30–70 µg/mL and 10–30 µg/mL, respectively and LOD and LOQ values were found to be 2.85 and 9.50 µg/mL for both 2,4-D and the herbicide. The sensitivity of the biosensor was calculated as 0.087 µA.mL / µg.cm^2 ^.This is the first study in literature by means of not only voltammetric detection of 2,4-D and DNA interaction but also the herbicide-DNA interaction at the surface of PGE based on the changes at the guanine signal.

## 1. Introduction

Synthetic auxins are a class of herbicides, and they are applied to control broadleaf weeds [1,2]. 2,4-dichlorophenoxyacetic acid (2,4-D) is chlorinated phenoxy herbicide [3] and one of the most commonly used auxinic herbicide [1,4,5], and it’s widely used in forests, rangelands, lawns, roadways, etc. [1,6,7]. Fast penetration through the roots or leaves occurs in its commercial form as the commercial ones are solved in water easily [4]. Due to its water solubility, it easily penetrates the underground water and often detected in surface water. Then, the use of 2,4-D becomes a contamination factor, exposition threats the aquatic life as well as vegetations [4]. 2,4-D has been classified as moderately hazardous (class II) based on its acute toxicity and slightly to moderately toxic (category II–III) by U.S. EPA [1,4]. It’s been reported that 2,4-D can be classified as harmful compound for aquatic life and it has long lasting harmful effects. Moreover, it’s reported that 2,4-D is an endocrine disruptive chemical [6]. The genotoxic effect of 2,4-D has been proven by in vivo and in vitro studies; it causes DNA damage [8] and strand breaks [6]. Thus, detection of 2,4-D and introducing new aspects for understanding of the interaction of DNA and 2,4-D have gained importance. 

Electrochemical biosensors provide detecting the target molecule not only specifically and sensitively but also practically. They are robust and portable analytic tools in a time-saving manner [9–14]. There are many reports in the literature for detection of 2,4-D by using electrochemical biosensors [15–24]. Most of them were focused on the detection of 2,4-D by using antibody based immunosensors [18,19] or molecularly imprinted polymer (MIP) based biosensors [15, 22–24]. As an example for immunosensor, gold electrode based impedimetric immunosensor was developed by Navratilova and Skladal [18] for detection of 2,4-D. Three type of gold electrode was tested by means of detection of 2,4-D. An MIP based photoelectrochemical biosensor was reported for detection of 2,4-D [15]. A titanium (Ti) substrate was used for fabrication of the biosensor; molecularly imprinting was performed at the surface of titanium dioxide (TiO_2_) and polypyrene (PPy) based polymer surface. 

There are some reports in the literature for detection of 2,4-D by using pencil graphite electrodes (PGEs) due to the advantages of them in comparison to conventional metal or carbon-based electrodes [22,24]. PGEs allow to design disposable, sensitive, selective, and cheap recognition platforms by using less complicated experimental procedures without extra polishing or cleaning steps [14,22,24–27]. Prusty and Brand [22] worked for the development of an MIP based capacitive biosensor for monitoring of 2,4-D. MIP was electropolymerized onto the surface of PGE, then the detection was performed. The selectivity of the biosensor was tested against other molecules. Another MIP modified PGE based electrochemical biosensor was introduced by Azadmehr and Zarei [24]. First, they modified at the surface of PGE by using chitosan and multiwalled carbon nanotube. They used this modified PGEs for the immobilization of both DNA and MIP. They found that DNA immobilization increased the sensitivity of the biosensor and immobilized both MIP and DNA for 2,4-D detection. They measured the oxidation current of Fe^2+^ for the detection of 2,4-D. 

Ahmadi and Bakhshandeh [28] studied the interaction of DNA and 2,4-D by using spectroscopic and voltammetric techniques. In the electrochemical part of the study, they used mercury drop electrode as the biosensor surface, electrodeposited 2,4-D onto the electrode and performed the interaction by adding DNA. They found that the binding mechanism of 2,4-D on double stranded DNA awas groove binding. Although there are some reports for detection of 2,4-D using PGEs or monitoring of the biointeraction of 2,4-D and DNA using HDME, there is no report in the literature for the label-free detection of the interaction between 2,4-D and DNA using disposable PGEs. At this point, it should be emphasized that PGEs allow to fabricate cheap, easy-to-use, reproducible, and sensitive biomonitoring platforms for recognition of nucleic acid targeted (bio)molecules [14,22,24–27].

Since the discovery of electroactive structure of nucleic acids [29], nucleic acid based biosensing platforms have been developed in combination with electrochemical techniques for the detection of different (bio)molecules such as drugs [26,30], toxins [27] proteins [31,32], target nucleic acids [33,34], which are biomarkers for diseases. DNA-targeted molecules can also be monitored by using nucleic acid immobilized surfaces and electrochemical techniques [26,27,30,35]. To monitor the effect of a DNA-targeted molecule in a label-free way, the change at the oxidation signal of the guanine base is evaluated. The guanine signal is measured at +1.0 V peak potential at carbon electrodes in acidic media. As an example, daunorubicin, which is an anticancer drug, caused the decrease at the guanine signal. This decrease changes the concentration of daunorubicin or the duration of the interaction process [26]. Monitoring of the guanine oxidation signal directly allows to develop label-free detection systems as well as to evaluate DNA damage induced by the (bio)molecules. Also, different types of (bio)sensors for detection of environmental pollutant were reported in the literature [36–45], and some of them were summarized in Table 1. 

**Table 1:** Biosensor platforms developed for the detection of different (bio)molecules and environmental pollutants. Abbreviations: Electrodes: LVN-PGE: Levan modified PGE, AuNP/PEDOT:PSS/GCE: Gold nanoparticle/poly (3, 4-ethylenedioxythiophene): polystyrenesulfonate (PEDOT:PSS)/glassy carbon electrode, AuE: Gold electrode, Fe3O4@ZIF-4: magnetic iron nanoparticle:zeolitic imidalozate, SWCNT-SubPc-Pc: single walled carbon nanotube-subphthalocyanine, Cu-SWCNT-Pc 3D: 2,3,9,10,16,17,23,24-Octakis (4-methyl-2,6-bis((prop-2-yn-1-yloxy)methyl)phenoxy) phthalocyaninato zinc(II) bearing sixteen terminal ethynyl groups attached to single-walled carbon nanotube, SWCNT-ZnPc: Asymmetric zinc (II) phthalocyanine (ZnPc) including three boron dipyrromethene (BODIPY) and one ethyloxy azido moieties decorated single walled carbon nanotube, Pt electrode: Platinum electrode, Methods: DPV: Differential pulse voltammetry, DPSV: Differential pulse stripping voltammetry, EIS: Electrochemical impedance spectroscopy, CV: Cyclic voltammetry.

Analyte	Electrode type	Method	Detection limit	Reference
Daunorubicin	LVN-PGE	DPV	510 nM	[26]
Fumonisin B1	PGE	EIS	3.69 ng/mL	[27]
p53 protein	AuNP/PEDOT:PSS/GCE	CV	0.09 ng/mL	[31]
Exosomal proteins	AuE	CV	1.66 × 104 particles/mL	[32]
Coumaric acid	Fe3O4@ZIF-4/GCE	DPV and CV	0.18 µM	[36]
Methyl parathion, acetamipridand chlorantraniliprole	GCE/SWCNT-SubPc-Pc	DPV	1.78 nM	[37]
Physostigmine	Cu-SWCNT-Pc 3D/GCE	DPV	53 nM	[38]
Methyl parathion, deltamethrin,chlorpyrifos and spinosad	SWCNT-ZnPc/GCE	DPV	1.49 nM	[39]
Quercetin	Polyglycine-GCE	DPSV and CV	0.39 µg/L	[40]
Ammonia	SWCNT) with 3-phenylcoumarin/Pt electrode	Ellipsometry	-	[41]

Herein, the author developed an electrochemical biosensor for monitoring of the interaction of 2,4-D and DNA. For this purpose, surface-confined interaction of DNA and 2,4-D was performed at the surface of disposable PGEs, then, the oxidation signal of guanine was measured. The experimental parameters were optimized, and the interaction was also evaluated in the presence of another herbicide, glyphosate, which is widely used in combination with 2,4-D for weed control [46,47]. The interaction was also tested in the presence of commercially available herbicide sample whose active molecule was 2,4-D. Until today, there is no report in the literature for the detection of the interaction of 2,4-D and DNA or the interaction of the herbicide and DNA at the surface of PGE based on the changes at the guanine signal. 

## 2. Materials and methods 

### 2.1. Apparatus

All electrochemical measurements were performed by using IVIUM Compactstat.e with IVIUM Release 4.951 software package (Holland). 

Pencil graphite electrode (PGE) as the working electrode, an Ag/AgCl/3M KCl as the reference electrode (BAS, Model RE-5B, W. Lafayette, USA) and a platinum wire as the auxiliary electrode were the elements of three-electrode system. Rotring Pencil model (Germany) was used to hold the graphite lead (Tombow 0.5 HB, Japan). A metallic wire was soldered to the metallic part to provide the electrical contact with the lead. Fourteen mm of the lead was held vertically, and the immersed portion of the lead into the solution was 10 mm for each measurement. 

### 2.2. Chemicals

Double stranded fish sperm DNA (DNA), 2,4-D, glyphosate (GLY), dimehthyl sulfoxide (DMSO), trizma hydrochloride (NH_2_C(CH_2_OH)_3_·HCl), ethylenediaminetetraaceticacid disodium salt dihydrate (C_10_H_14_N_2_Na_2_O_8_·2H_2_O), potassiumhexacyanoferrate(II) trihydrate (K_4_Fe(CN)_6_·3H_2_O), potassiumhexacyanoferrate(III) (K_3_Fe(CN)_6_), potassium chloride (KCl), di-potassiumhydrogen phosphate (K_2_HPO_4_), potassiumdihydrogen phosphate (KH_2_PO_4_), acetic acid (CH_3_CO_2_H) and sodium chloride (NaCl) were purchased from Sigma-Aldrich (Sigma-Aldrich Corp., St. Louis, MO, USA). DNA stock solution was prepared at 1000 µg/mL concentration level in Tris-EDTA buffer solution (10 mM Tris-HCl, 1 mM EDTA, pH 8.00) and kept frozen. DNA was diluted in 0.50 M acetate buffer containing 20 mM NaCl (pH 4.80, ABS), and 2,4-D stock solution was prepared DMSO as 1000 µg/mL. The diluted solutions of 2,4-D were prepared with 50 mM phosphate buffer solution (PBS; pH 7.40). A total of 1000 µg/mL GLY stock solution was prepared using 50 mM PBS (pH 7.40) and the dilutions of GLY was performed with 50 mM PBS (pH 7.40). 

The herbicide whose formulation was dimethyl amine salt of 2,4-D was purchased from local market. Its stock solution was 500.000 µg/mL. The diluted solutions of the herbicide was prepared in PBS (pH 7.40).

Other chemicals were of analytical reagent grade and were supplied from Sigma-Aldrich and Merck. Ultrapure water was used in other stock solutions.

### 2.3. Procedure 

**Figure 1:**
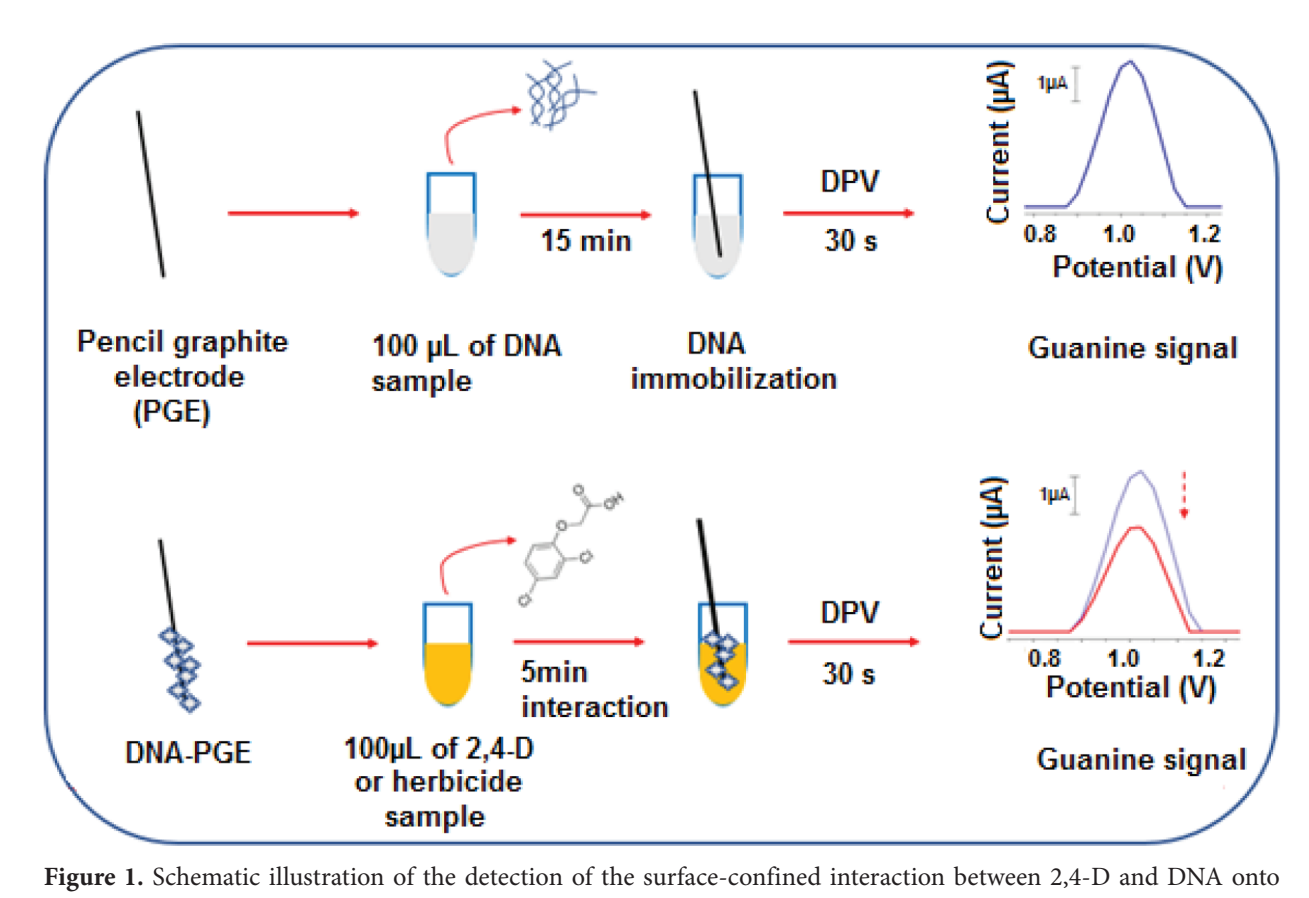
Schematic illustration of the detection of the surface-confined interaction between 2,4-D and DNA onto disposable PGE surface.

The experimental steps of the development of DNA immobilized PGE and the interaction of 2,4-D and DNA at the surface of PGE was represented in Figure 1. 

#### 2.3.1.Preparation of DNA immobilized PGE

Before use, electrochemical pretreatment of PGEs was done by applying a potential of +1.40 V for 30 s in ABS (pH 4.80). DNA immobilized at the surface of PGE by using passive adsorption [26]. 100 µL of required amount of DNA was immobilized at the surface of PGE during required immobilization time. Then, the PGEs were washed with ABS (pH 4.80) to remove unspecific binding of DNA molecules. 

#### 2.3.2. The interaction of DNA and 2,4-D at the surface of PGE

100 µL of 30 µg/mL DNA was immobilized at the surface of PGE during 15 min. Unspecific DNA binding was eliminated by washing the DNA immobilized PGEs in ABS (pH 4.80). DNA immobilized electrodes were immersed in 100 µL of required amount of 2,4-D during required interaction time. Then, all electrodes were washed in PBS (pH 7.40).

#### 2.3.3. The interaction of DNA and GLY or the herbicide sample 

For the interaction of DNA and GLY, 30µg/mL DNA immobilized PGEs were immersed into the vials containing 100 µL of 70 µg/mL GLY during 5 min. Then, the electrodes were washed in PBS (pH 7.40). 

For the interaction of DNA and the herbicide, 30µg/mL DNA immobilized PGEs were immersed into the vials containing 100 µL of required amount of herbicide sample during 5 min. After then, all electrodes were washed in PBS (pH 7.40).

#### 2.3.4. Voltammetric measurements

Before/after interaction process, the guanine oxidation signal was observed by using differential pulse voltammetry (DPV) technique. DPV measurements were performed in ABS (pH 4.80) between the potential of +0.75 V and +1.25 V at a pulse amplitude of 50 mV and scan rate of 50 mV/s.

CV measurements were performed in a redox probe solution of 2.00 mM K_3_[Fe(CN)_6_]/K_4_[Fe(CN)_6_] (1:1) prepared in 0.10M KCl using the potential range from −0.45 V to +1.20 V with the scan rate as 50 mV/s. 

The behaviour of the electron transfer was determined by calculating peak-to-peak separation (ΔE_p_) values based on Equation (1) as below: 

E_a_-E_c _= 0.059/n, 

where E_a_ and E_c_ represent the peak potentials of anodic and cathodic peak currents, respectively.

The effective surface area (A_eff_) values were calculated according to the Randles–Sevcik Eq. (Equation (2)) [48] using I_a_ values and represented in Table 2. The transferred electron number is n, D is the diffusion coefficient of K_3_[Fe(CN)_6_] (7.6 × 10^−6^ cm^2^ s^−1^), C is the concentration of K_3_[Fe(CN)_6_] in this equation. 

Equation (2)ip=2.69105n3/2AeffD1/2Cv1/2

**Table 2:** The elemental concentrations for carbon (C), oxygen (O), nitrogen (N), and phosphorus (P) atoms obtained by PGE (A), after the immobilization of 30 µg/mL DNA at the PGE (B) and after the interaction of 70 µg/mL 2,4-D and 30 µg/mL DNA at PGE (C).

	Element	Weight %	Atomic%
A	C	62.74	69.17
	N	0.01	0.01
	O	37.24	30.82
	P	0.00	0.00
B	C	96.46	97.93
	N	0.63	0.54
	O	1.02	0.78
	P	1.90	0.75
C	C	98.32	98.70
	N	0.42	0.36
	O	1.23	0.93
	P	0.03	0.01

#### 2.3.5.Impedimetric measurements

Impedimetric measurements were done in a redox probe 2.50 mM K_3_[Fe(CN)_6_]/K_4_[Fe(CN)_6_] (1:1) prepared in 0.10 M KCl. The impedance was measured in the frequency range from 100 mHz to 100 kHz at a potential of + 0.23 V with a sinusoidal signal of 10 mV. The frequency interval divided into 98 logarithmically equidistant measure points. Randles circuit was used as the equivalent circuit model for the purpose of fitting of the impedance data. R_ct _represents the respective semicircle diameter corresponds to the charge-transfer resistance occurred at the electrode–electrolyte interface, and the other parameters are the solutions resistance (R_s_), the capacitance (Q), which is related to the space charge capacitance occurred between the electrode–electrolyte interface, and Warburg impedance (W) due to mass transfer to the electrode surface. 

All experimental steps were performed in room temperature. 

## 3. Results and discussion

**Figure 2:**
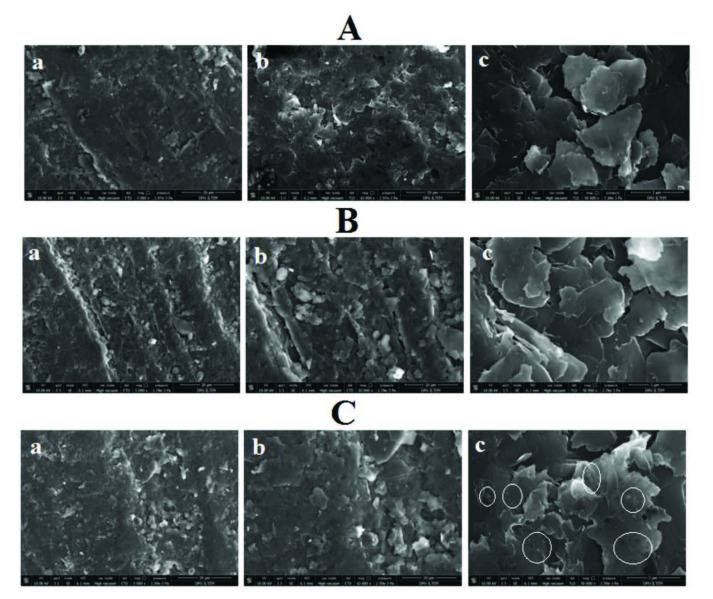
SEM images obtained by the PGE (A), after the immobilization of 30 μg/mL DNA at the PGE (B) and after the interaction of 70 μg/mL 2,4-D and 30 μg/mL DNA at the PGE (C). The acceleration voltage was 10.0 kV, the resolutions were 5000  (the scale was 20 μm) (a), 10000  (the scale was 10 μm) (b) and 50000  (the scale was 2 μm) (c).

Firstly, microscopic characterization of DNA modification and 2,4-D and DNA interaction was investigated using Scanning Electron Microscopy (SEM) technique (Figure 2). Immobilization of 30 µg/mL DNA at PGE (Figure 2A,a and Figure 2A,b) could be clearly seen (Figure 2B,a and Figure 2B,b) by stacking of DNA molecules at the multi-layered graphite surface. After the interaction of 70 µg/mL 2,4-D and 30 µg/mL DNA at PGE, the graphite surface could be partially covered (Figure 2C,b), and some bright spots onto the layers could be observed (Figure 2C,c, circled ones). 

**Figure 3:**
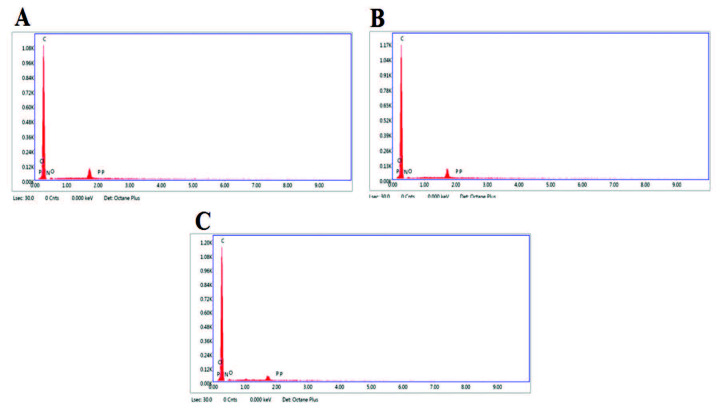
EDX spectra obtained by the PGE (A), after the immobilization of 30 μg/mL DNA at the PGE (B) and after the interaction of 70 μg/mL 2,4-D and 30 μg/mL DNA at the PGE (C).

Energy dispersive X-ray spectroscopy (EDX) spectras and the elemental concentrations of C, O, N, and P atoms were given in Figure 3 and Table 2. The immobilization of DNA resulted the increase at the weight% values of C, N, P (Figure 3B) compared to the bare electrode (Figure 3A). This result could be attributed that the introduction of nucleotides which have nitrogenous base and phosphate backbone of DNA caused the increase at these atoms. After the interaction of 2,4-D and DNA at the surface of PGE, weight% values of C and O atoms increased as a result of introduction of 2,4-D molecules rich in C and O atoms (Figure 3C). Microscopic results indicated that the immobilization of DNA and the interaction between DNA and 2,4-D could be successfully achieved at the surface of disposable PGEs. 

**Figure 4:**
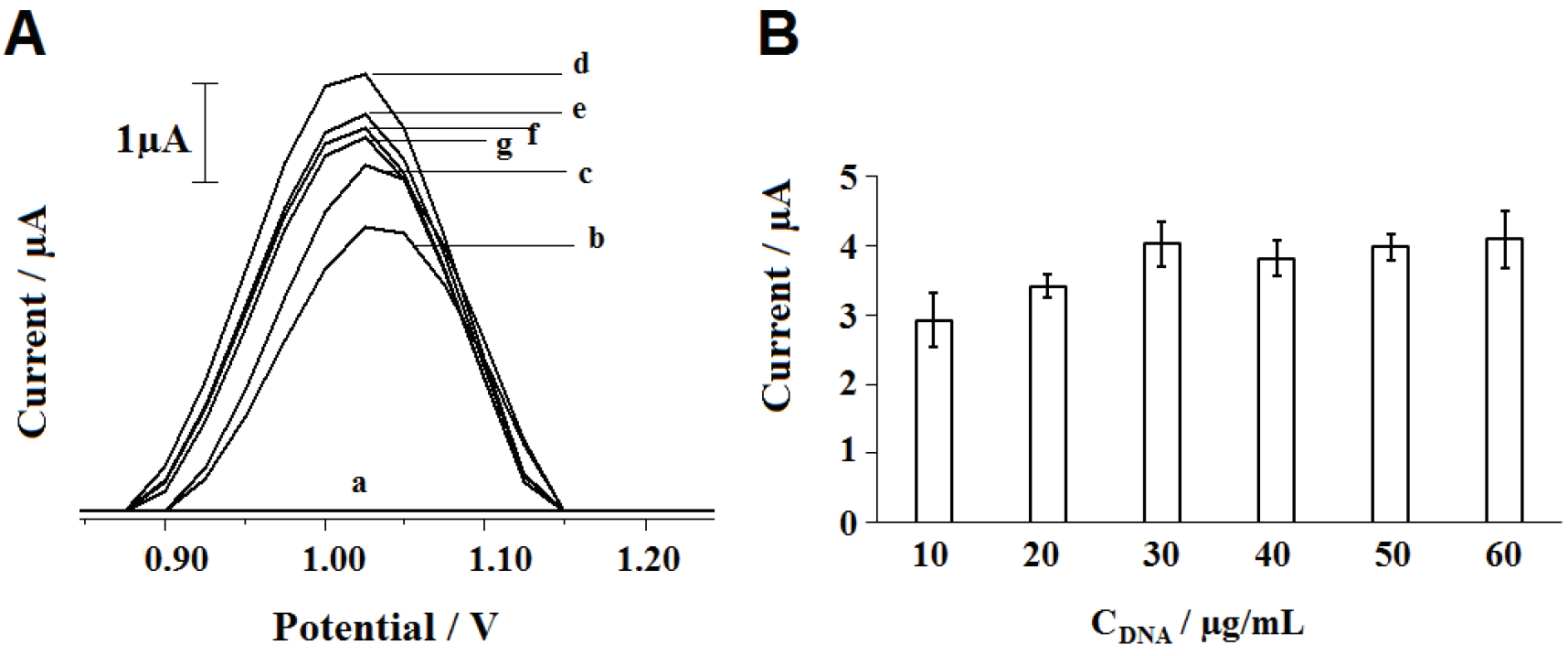
T he effect of DNA concentration onto the guanine oxidation signal obtained at +1.025 V by using DPV technique. Voltammograms (A) obtained by bare PGE (a), 10 (b), 20 (c), 30 (d), 40 (e), 50 (f) and 60 (g) μg/mL DNA immobilized PGE. Histograms (B) representing the average guanine signals by using 10–60 μg/mL DNA immobilized PGE (n = 3)

For electrochemical monitoring, the experimental conditions were optimized. First, the effect of DNA concentration upon the biosensor response was investigated. For this purpose, 10-60 µg/mL DNA was immobilized at the surface of PGE during 10 min. After then, DPV measurements were performed. The voltammograms of the guanine oxidation signals measured at + 1.025 V and the average guanine signals obtained at each DNA concentration level were represented in Figure 4. Guanine signal increased till 30 µg/mL, then decreased at 40 µg/mL concentration level and stabilized while DNA concentration increased. The highest guanine signal could be obtained by using 30 µg/mL DNA as 4.02 ± 0.32 µA with the relative standard deviation % (RSD%) as 7.96% (n = 3) (Figure 4A,d and Figure 4B). 30 µg/mL concentration was chosen as optimum for DNA immobilization. 

**Figure 5:**
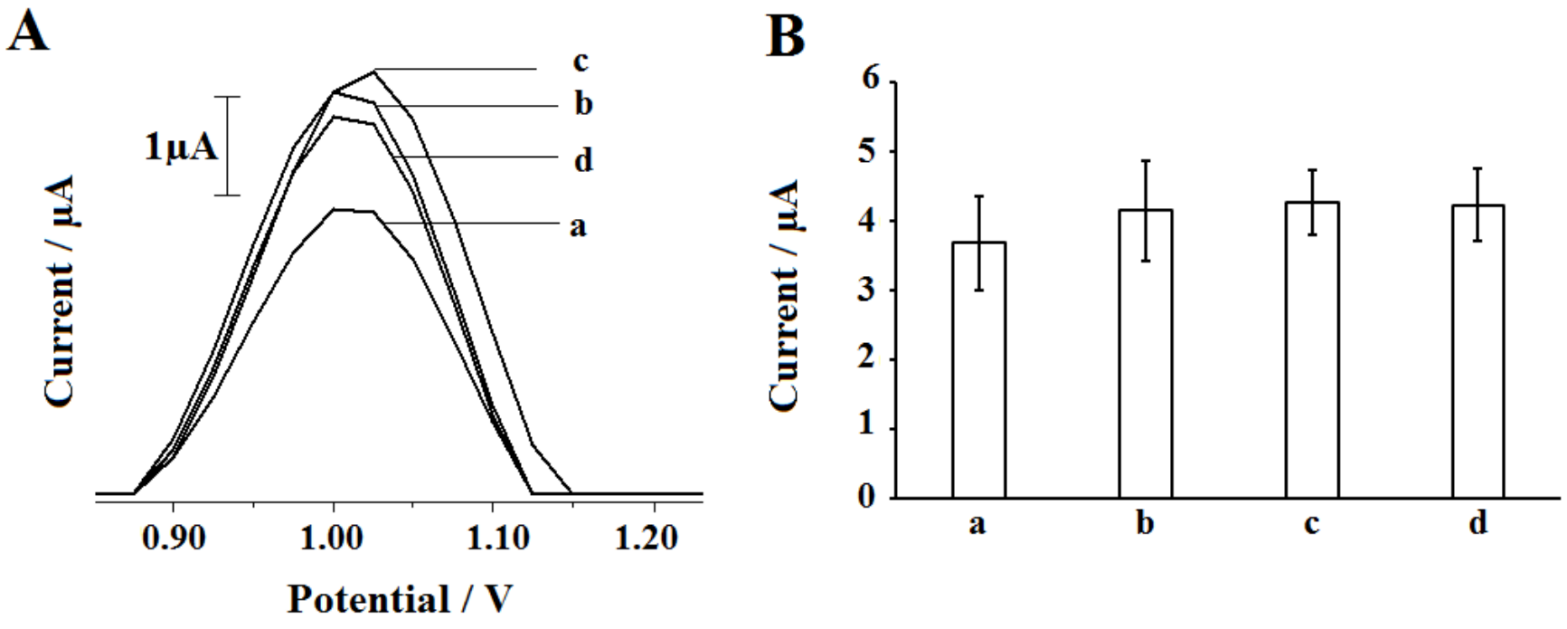
The effect of DNA immobilization time onto the guanine signal. Voltammograms representing the guanine signals (A) and histograms (B) representing the average guanine signals (n = 3) obtained after 30 μg/mL DNA immobilization at the surface of PGE using 5 (a), 10 (b), 15 (c) and 20 (d) min immobilization time.

The effect of DNA immobilization time was then investigated by using 5–20 min immobilization time (Figure 5). The highest guanine signal could be measured after 15 min immobilization of 30 µg/mL DNA at PGE surface (Figure 5A,B,c) as 4.26 ± 0.46 µA (RSD % = 10.90, n = 3). 15 min was chosen as optimum for DNA immobilization time. 

**Figure 6:**
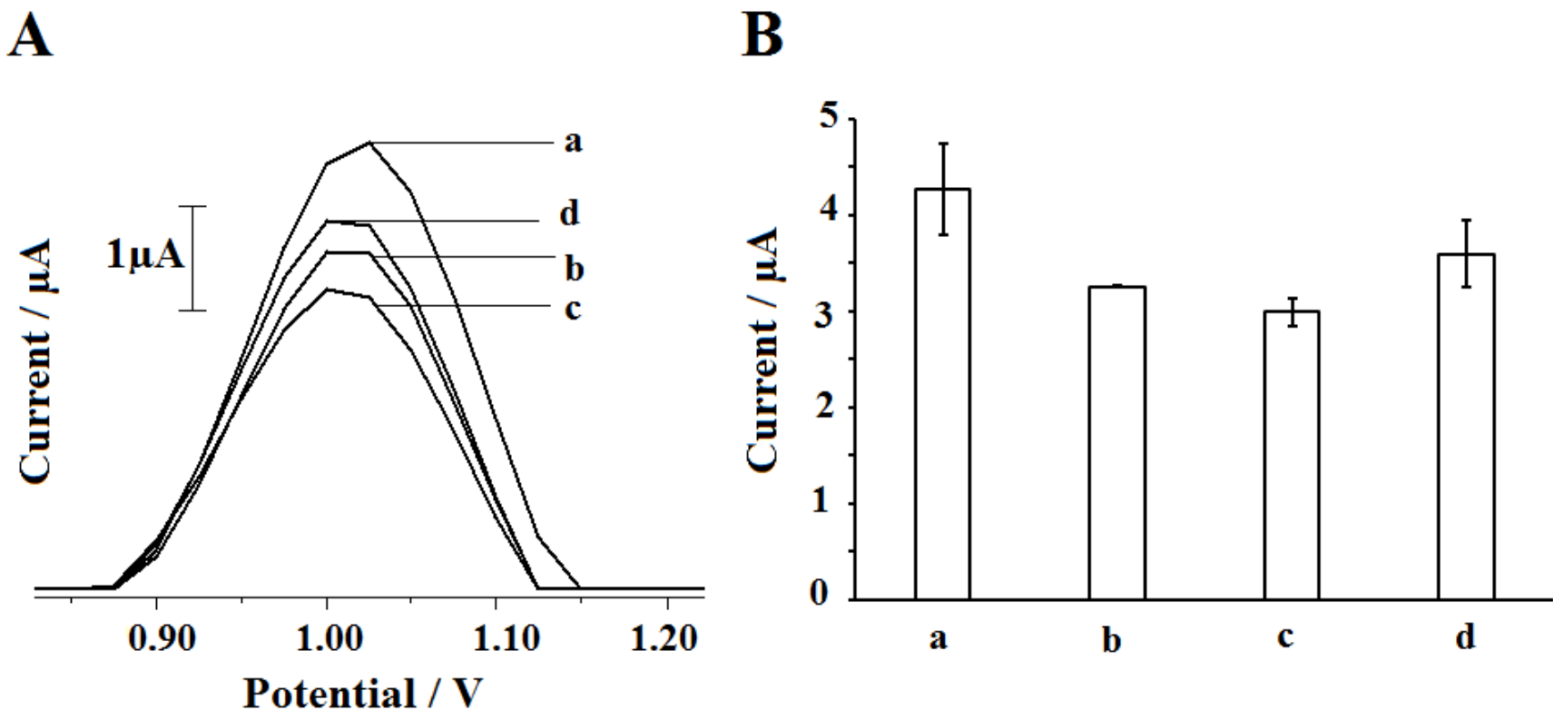
Voltammograms representing guanine signals (A) and histograms representing the average guanine signals (n=3) (B) obtained before interaction (a), after the interaction between 30 μg/mL DNA and 50 μg/mL 2,4-D during 3 (b), 5 (c) and 10 (d) min at the surface of the PGE.

After the optimization of DNA immobilization conditions, the surface-confined interaction of DNA and 2,4-D was studied. First, effect of interaction time was evaluated (Figure 6). The interaction of 50 µg/mL 2,4-D and 30 µg/mL DNA was performed during 3, 5 and 10 min, then, decreases at the guanine signal were observed after each interaction time. 2,4-D had groove-binding behavior at pH 7.30 [28], then caused to decrease at guanine signal after the interaction. The oxidation of 2,4-D was also studied and any signal was observed at the potential range from +0.75 V to 1.25 V from 2,4-D. The decrease ratios were evaluated by means of interaction process. There were 23.82%, 29.89%, and 15.73% decreases at the guanine signal after 3 min, 5 min, and 10 min interaction, respectively and 5 min was chosen as optimum interaction time. The average guanine signal could be obtained as 2.99 ± 0.15 µA (RSD%=4.85%, n=3) after 5 min interaction (Figure 6B,c). 

**Figure 7:**
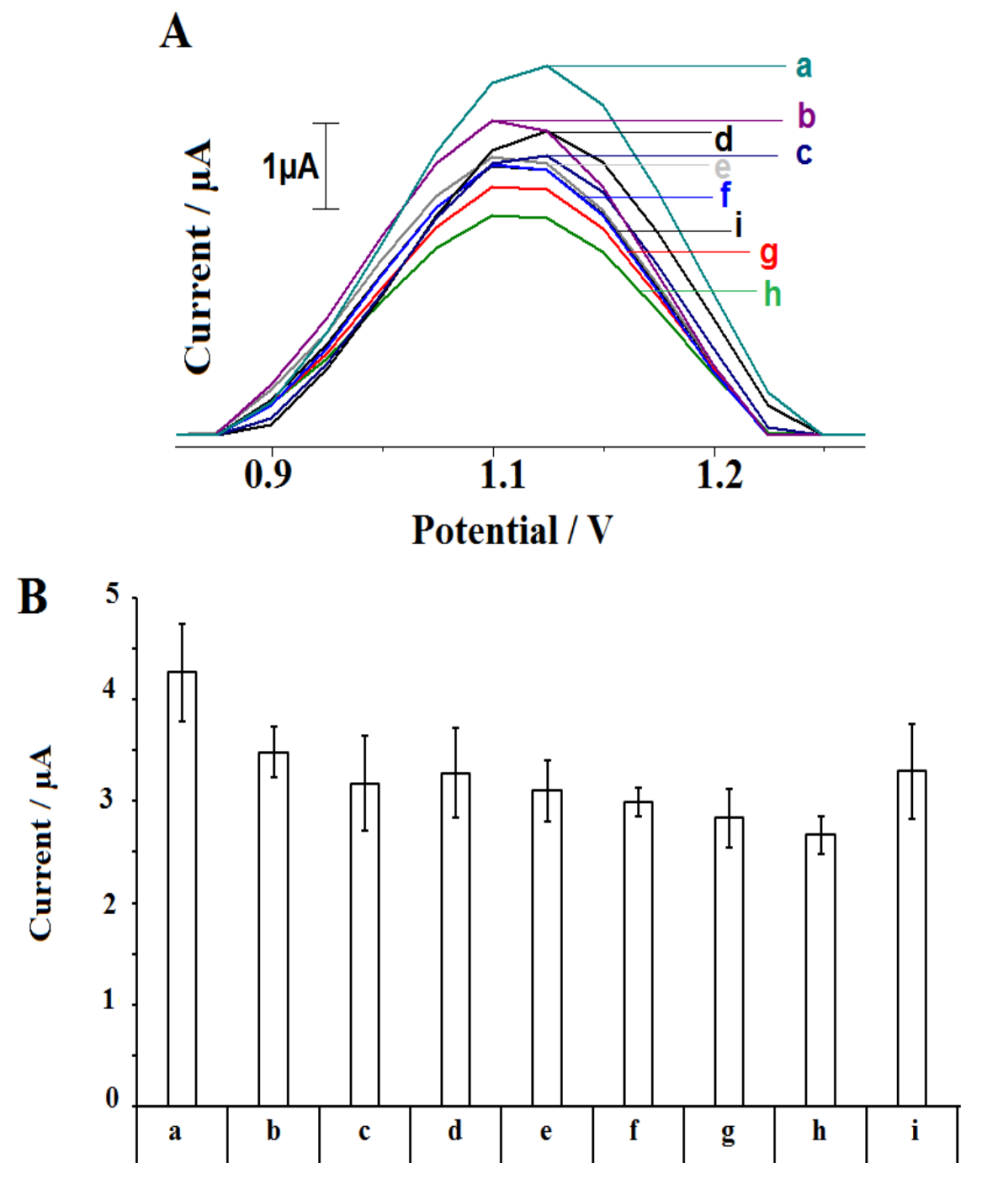
Voltammograms (A) and the average guanine signals (B) obtained before (a) and after interaction between 30 μg/mL DNA and 10 (b), 20 (c), 30 (d), 40 (e), 50 (f), 60 (g), 70 (h) and 80 (i) μg/mL 2,4-D during 5 min at the surface of the PGE (n=3).

**Figure 8:**
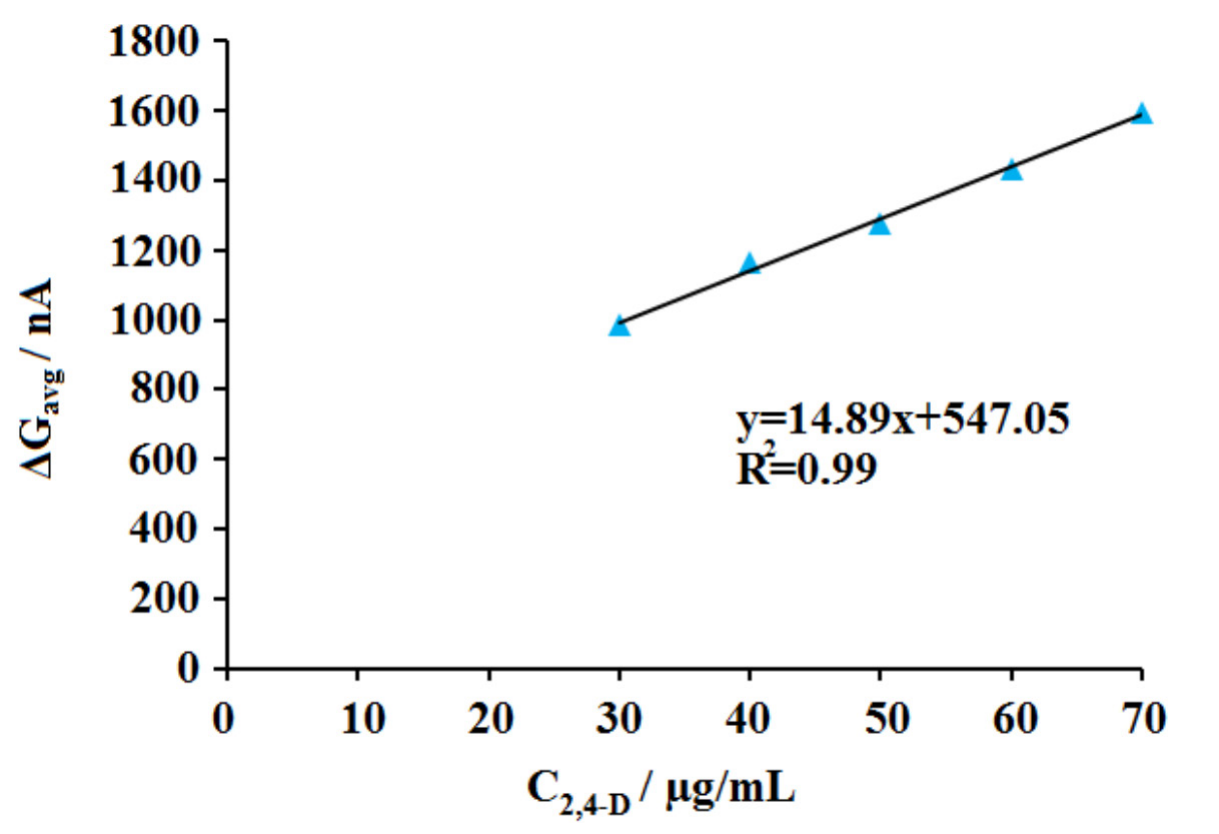
Line graph representing the differences between the average guanine signals (ΔGavg) obtained before and after interaction of 30 μg/mL DNA and 30-70 μg/mL 2,4-D onto the surface of the PGE (n = 3).

The effect of 2,4-D concentration upon the interaction was then investigated (Figure 7). The interaction of 30 µg/mL DNA and 2,4-D at different concentration level from 10 to 80 µg/mL was performed. The guanine signal was monitored before/after interaction process. Increasing dimunition at the average guanine signal (n = 3) could be obtained till 70 µg/mL, and the highest decrease at the guanine signal was monitored at this concentration level of 2,4-D as 37.46% (Figure 7h). The average guanine signal after the surface-confined interaction between 30 µg/mL DNA and 70 µg/mL 2,4-D at the surface of PGE could be obtained as 2.69 ± 0.18 µA with the RSD%= 6.90% by three repetitive measurements. Also, the decrease at the guanine signal had linear behaviour. The discrimination (ΔG_avg_) between the average guanine signals (n = 3) obtained before (G1_avg_) and after (G2_avg_) interaction process was estimated using Equation (3) for each concentration of 2,4-D at 10-70 µg/mL concentration level. A calibration graph could be obtained based on ΔG_avg_ values (Figure 8). The detection limit (DL) of 2,4-D based on the biosensor response obtained by the DNA damage induced by 2,4-D was calculated using according to the method described by Miller and Miller [49] and was found to be 2.85 μg/mL. 

Equation (3)ΔGavg=G1avg-G2avg

**Figure 9:**
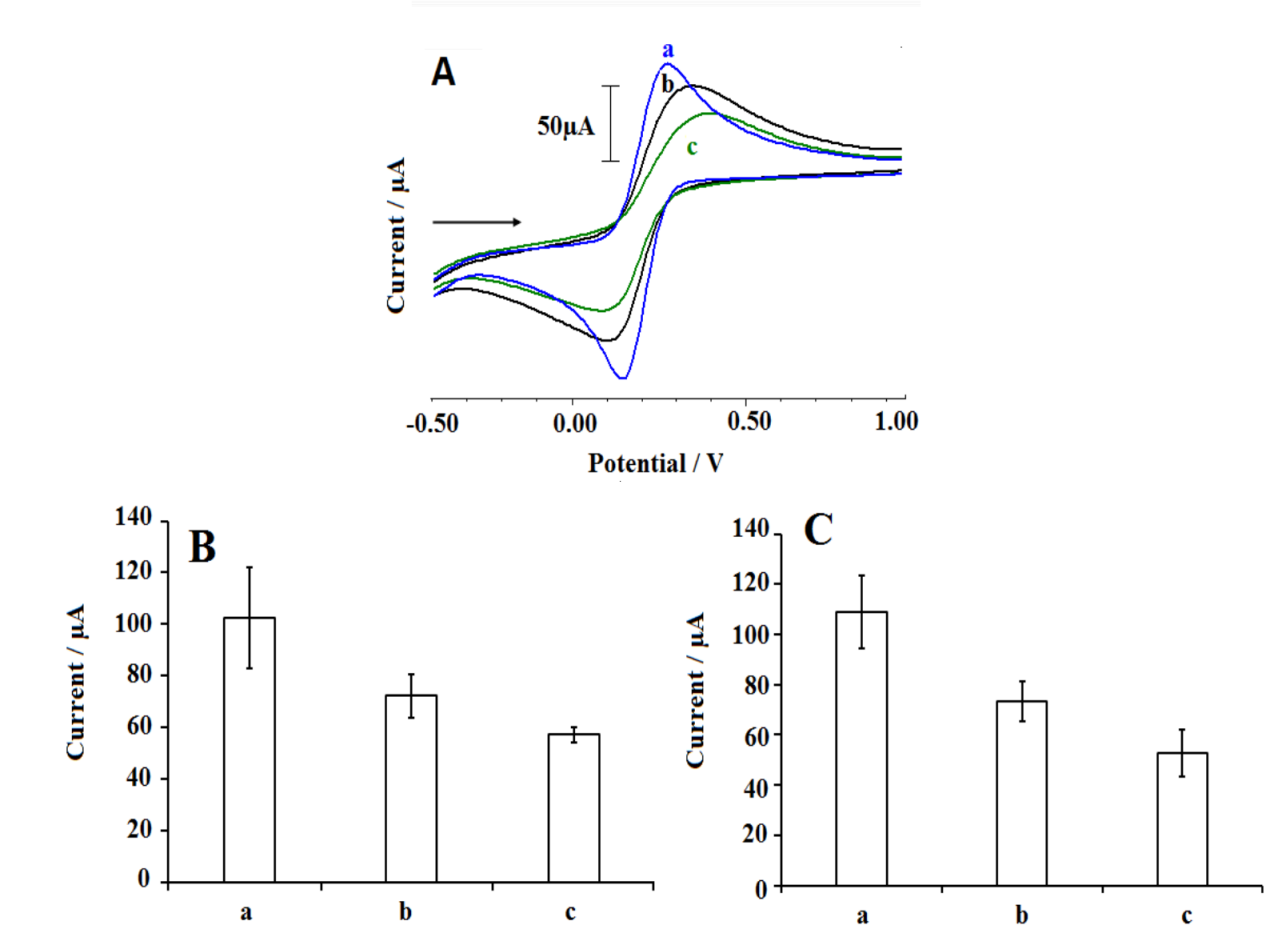
Cyclic voltammograms (A) obtained by PGE (a), 30 μg/mL DNA immobilized PGE (b) and after interaction between 30 μg/mL DNA and 70 μg/mL 2,4-D at the surface of the PGE (c). Average anodic (Ia) (B) and cathodic (Ic) (C) peak currents and obtained by PGE (a), 30 μg/mL DNA immobilized PGE (b) and after interaction between 30 μg/mL DNA and 70 μg/mL 2,4-D at the surface of PGE (c) (n = 3). 2 mMK3[Fe(CN)6]/K4[Fe(CN)6] (1:1) containing 0.10M KCl was used as supporting electrolyte solution.

The electrochemical characterization of the interaction mechanism occurred between DNA and 2,4-D under optimum conditions was furtherly studied by using CV and EIS, which are powerful techniques for interpretation and evaluation of the changes at the electrode/electrolyte interface. Figure 9 represents the voltammograms (Figure 9A), the average anodic (I_a_) (Figure 9B) and cathodic (I_c_) (Figure 9C) peak currents obtained by using CV technique performed before/after each experimental step. After the immobilization of DNA at the surface of PGE, average I_a_ and I_c_ values (n = 3) decreased respectively 29.59% and 32.78% due to the repulsive interaction between negatively charged phosphate backbone of DNA and Fe(CN)_6_]^3-/4-^ at PGE surface [50,51]. The average I_a_ and I_c_ values also decreased by 20.91% and 28.21%, respectively after the interaction of DNA at 2,4-D. These decreases obtained after the surface-confined interaction of DNA and 2,4-D could be attributed that the prevention of the electron transfer due to negative structure of 2,4-D [28]. The changes at the average anodic (Q_a_) and cathodic (Q_c_) charge values had similar behaviours with the changes at the I_a_ and I_c_ obtained after DNA immobilization or the interaction of 2,4-D and DNA. There were 9.44% and 10.47% decreases at the average Q_a_ and Q_c_ values obtained after the DNA immobilization at the surface of PGE, respectively. 22.08% and 26.42% decreases at the average Q_a_ and Q_c_ values could be monitored after the interaction between DNA and 2,4-D, respectively. The average I_a_, I_c_, Q_a_ and Q_c_ values were given in Table 3. 

**Table 3:** The average Ia, Ic, Qa and Qc values and ΔEp and Aeff values obtained before/after 30 µg/mL DNA immobilization at the surface of PGE and before/after interaction of 30 µg/mL DNA and 70 µg/mL 2,4-D at the surface of PGE.

	A	B	C
Ia/µA	102.49 ± 19.71	72.16 ± 8.34	57.07 ± 2.90
Ic/µA	108.84 ± 14.65	73.22 ± 8.07	52.56 ± 9.43
Qa/C*10–4	6.28 ± 0.67	5.69 ± 0.60	4.43 ± 0.19
Qc/C*10–4	5.06 ± 0.48	4.53 ± 0.45	3.33 ± 0.42
ΔEp/mV	130	270	310
Aeff/cm2	0.309	0.218	0.172

ΔE_p_ values and the effective electrode surface areas (A_eff_) of PGE, DNA immobilized PGE, the PGE with the interaction of DNA and 2,4-D onto its surface were calculated (Table 3). The ΔE_p _values showed that the DNA immobilization and the interaction process provided that the electrochemical reaction of K_3_[Fe(CN)_6_] ions at the electrode surface was irreversible [52–54]. The A_eff _values changed similar to the changes observed at the I_a_ values. After the immobilization of DNA molecules, the A_eff _value decreased as a result of the coverage of the graphite surface. The negatively charged DNA molecules caused to decrease at the I_a_ value, then the A_eff _value decreased. After the biointeraction between DNA and 2,4-D, the A_eff _value decreased due to decrease at the I_a_ value. 

**Figure 10:**
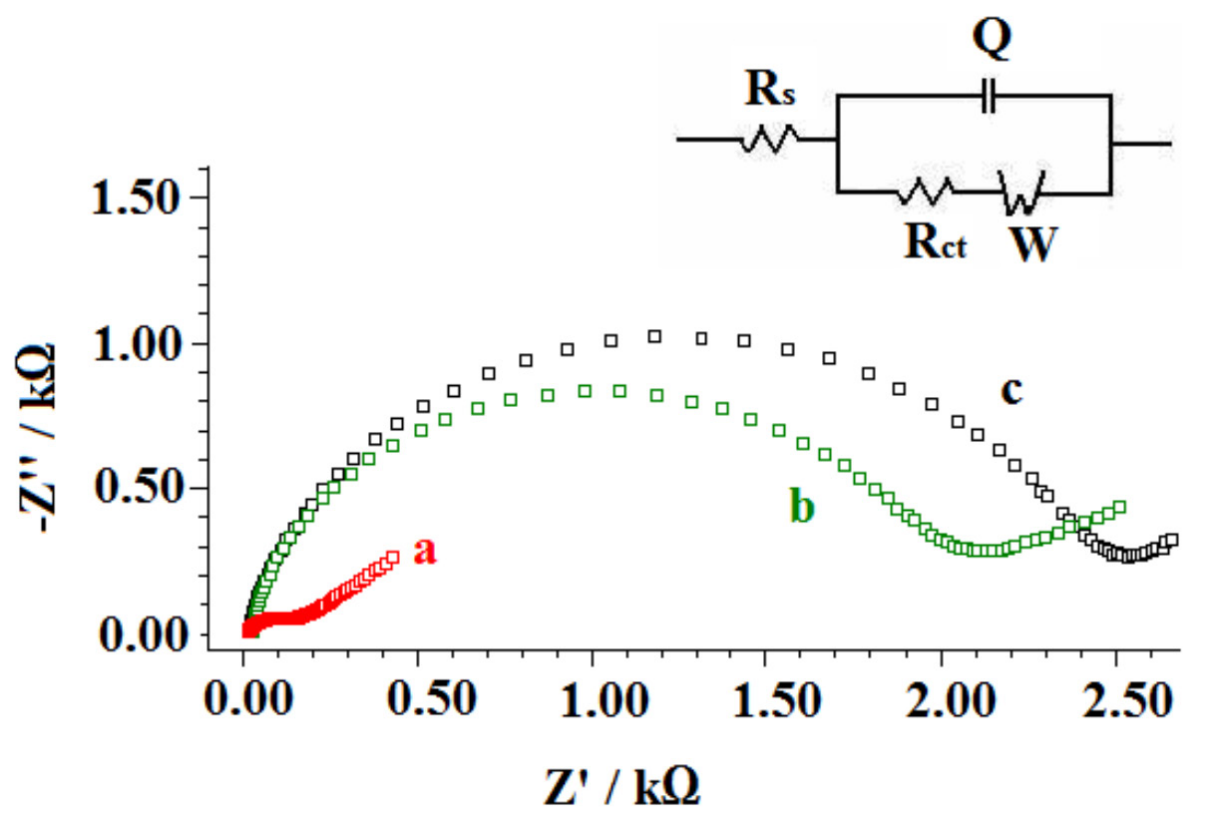
Nyquist diagrams obtained (a) by the PGE, by 30 μg/mL DNA immobilized PGE (b) after (c) interaction between 30 μg/mL DNA and 70 μg/ mL 2,4-D at the surface of the PGE (c).

Figure 10 represents the Nyquist diagrams obtained by using EIS technique. DNA immobilization at the surface of PGE introduced negative behaviour at the electrode surface due to the phosphate backbone of DNA, then the electron transfer between the negatively charged redox probe and the electrode surface was partially blocked. This hinderic effect caused the increase at the charge transfer value (R_ct_) [51,55] and 12.15 fold increased R_ct _value was measured (Figure 10b). The R_ct_ value also increased after the interaction between 2,4-D and DNA (Figure 10c). The electrode surface became more negative due to the introduction of negatively charged 2,4-D structure [51], and it caused to the repulsive interaction between the redox probe and electrode surface. The R_ct_ values were 146 Ohm, 1920 Ohm and 2373 Ohm by using PGE, DNA immobilized PGE, PGE with the interaction of DNA and 2,4-D onto its surface, respectively. The appearent fractional coverage (Q_R_^IS^) values of the DNA immobilized PGE and PGE with the interaction of DNA and 2,4-D onto its surface were calculated using Equation (4) [56] as 0.92 and 0.19, respectively. 

Equation (4)QISR=1-Rct1/Rct2

The cyclic voltammetric and impedimetric results were consistent with each other. Moreover, the microscopic and electrochemical characterizations were an agreement which indicated that the surface-confined interaction of DNA and 2,4-D could be succesfully achieved.

**Figure 11:**
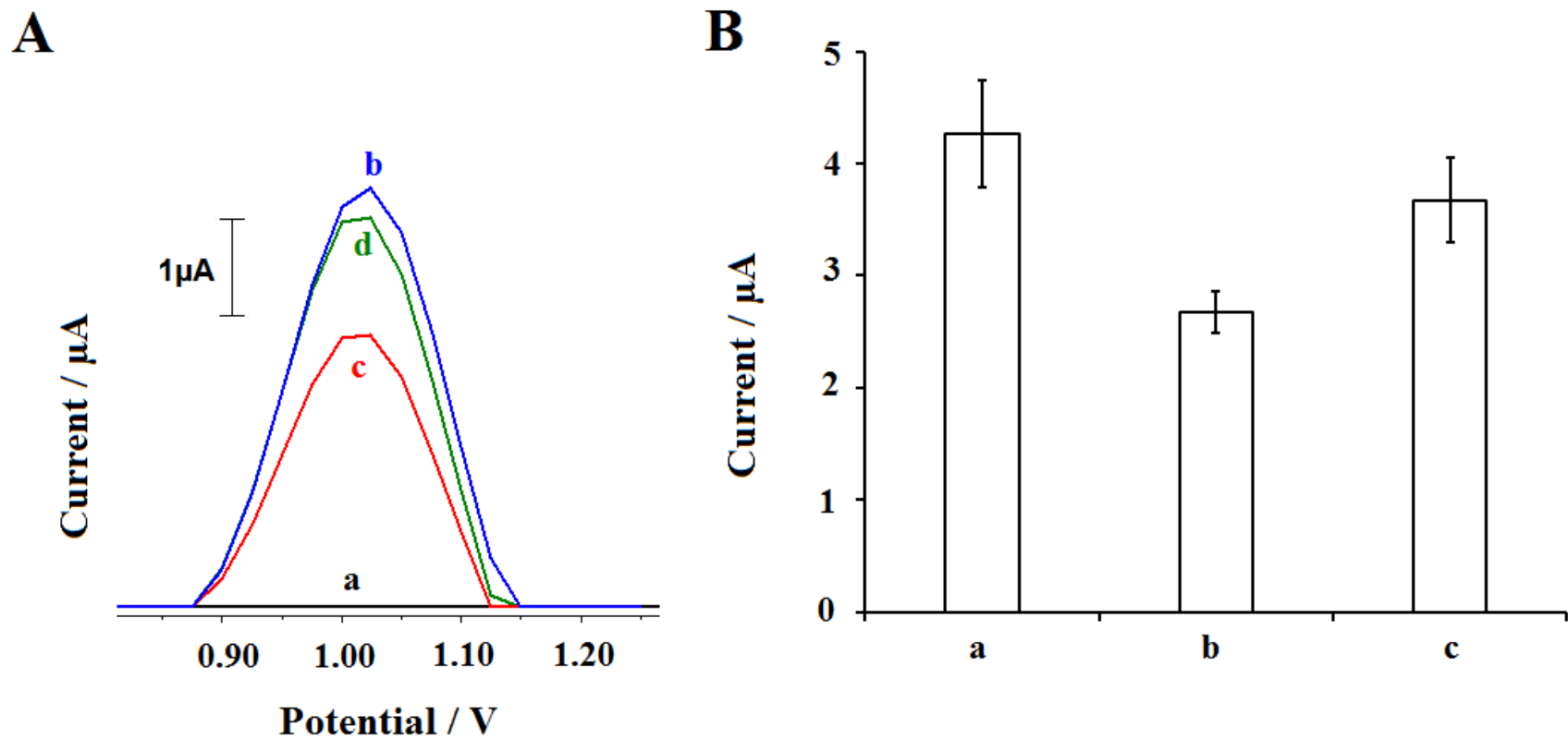
Voltammograms (A) of control signals (a) of 2,4-D or GLY the guanine signal obtained before (b) and after the interaction of 70 μg/mL 2,4-D (c) or GLY and 30 μg/mL DNA at the surface of PGE. The histograms representing the average guanine signals obtained before (a) and after the interaction of 70 μg/mL 2,4-D (b) or GLY (c) and 30 μg/mL DNA (n = 3).

In the next step of the study, the interaction process was investigated in the presence of a different pesticide, GLY under optimum conditions (Figure 11). It is known that the mixture of 2,4-D and GLY has been widely used for weed control [46,47]. Thus, the effect of 2,4-D onto DNA was compared to the one occurred in the presence of GLY. After the interaction between DNA and 2,4-D or GLY, the average guanine signals were obtained as 2.67 ± 0.18 µA (Figure 11A,c and Figure 11B,b) and 3.67 ± 0.38 µA (Figure 11A,d and Fig. 11B,c), respectively. There were 37.46% and 13.89% decreases at the guanine signals after the interaction of DNA and 2,4-D or GLY respectively. Both 2,4-D and GLY caused the decrease at the guanine signal, but higher decrease could be monitored after the interaction of DNA and 2,4-D. This result is in consistency with the result reported by Peterson and Hulting [57] who showed the phytotoxic effect of 2,4-D 400 times higher than GLY. The recognition system reported herein allowed to compare the genotoxic effect of 2,4-D against GLY at the concentration level of 70 µg/mL. 

**Figure 12:**
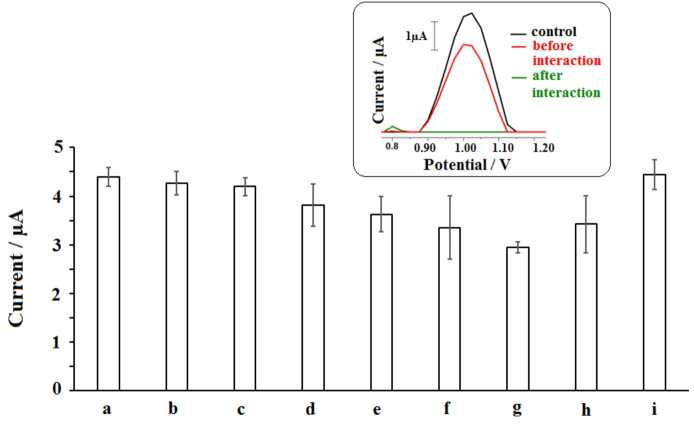
The average guanine signals obtained before (a) and after interaction between 30 μg/mL DNA and 5 (b), 10 (c), 15 (d), 20 (e), 25 (f), 30 (g), 35 (h) and 40 (i) μg/mL herbicide during 5 min at the surface of the PGE (n = 3). Inset: Voltammograms representing the guanine signals obtained before and after interaction between 30 μg/mL DNA and 30 μg/mL herbicide at the surface of the PGE.

**Figure 13:**
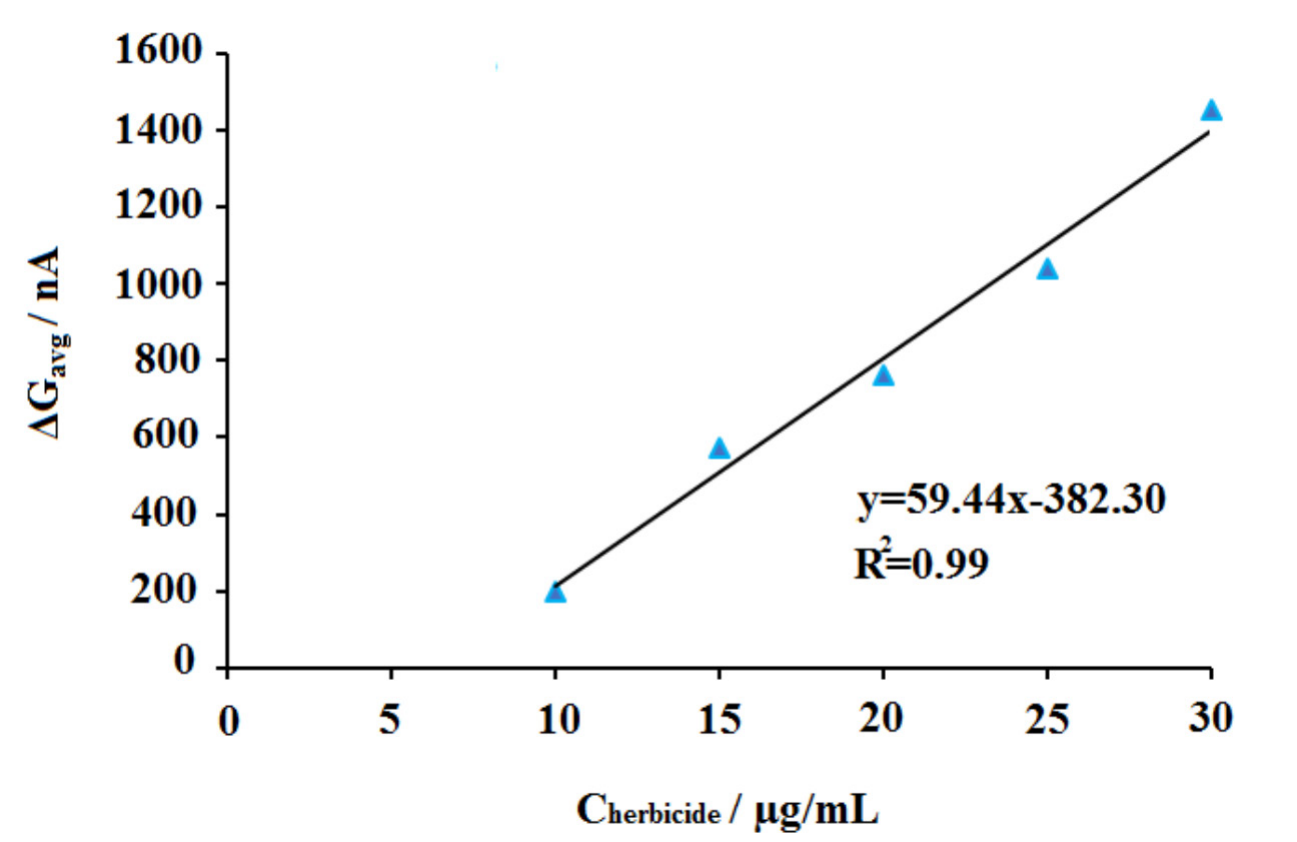
Line graph representing the differences between the average guanine signals (ΔGavg) obtained before and after interaction of 30 μg/mL DNA and 10–30 μg/mL herbicide onto the surface of PGE (n = 3).

The analysis of the interaction of DNA and the herbicide was tested in the last part of the study (Figure 12) to show whether the interaction occurred in a real herbicide sample or not. For this purpose, commercially available herbicide, whose active molecule was 2,4-D, was purchased and diluted at the concentration level of 5-40 µL (Figure 12b to i) and the interaction of DNA and the herbicide samples were performed at the surface of PGE. The decrease at the average guanine signal (n = 3) could be achieved after the interaction of DNA and the herbicide till 30 µg/mL. Similar to the results obtained by the interaction of DNA and 2,4-D (Figure 8), the decrease at the guanine signal had a linear behaviour, and a calibration graph could be obtained at 10-30 µg/mL concentration level (Figure. 13). The DL of 2,4-D based on the biosensor response of the DNA damage induced by the herbicide was calculated using the ΔG_avg_ values and Equation (3) and found to be 2.85 µg/mL [49]. The same DL could be obtained in the presence of 2,4-D or herbicide. This result showed not only the interaction between DNA and 2,4-D or herbicide was succesfully performed at the surface of PGE but also the robustness and accuracy of the developed biosensor. Although the DLs were higher than the DLs reported in the other studies [15;20-24], the biosensor platform developed within the scope of this study for monitoring of the interaction of 2,4-D and DNA has important advantages, such as being cheap, practical, and labor-friendly by using disposable graphite leads. Development of the biosensor was not required complicated experimental steps. The interaction could be monitored in only 20.5 min (including measurement) by using the voltammetric biosensor. Moreover, this is the first study in the literature by means of label-free detection of interaction of 2,4-D or the herbicide and DNA at PGE surface. 

Arcaute et al. [6] reported the LC_50 96h _valueas 1008mg/L 2,4-D for neotropical fish *Cnesterodon decemmaculatus.* In another study of this group, it was reported that exposure to 2.5% and 5% of the LC50_96h_ values (equals to 25.20 and 50.40 mg/L of 2,4-D) caused an increase in the frequencies of breaks in DNA of C. *decemmaculatus* . The concentration ranges of 2,4-D and the herbicide were quite lower than the ones reported by Arcaute et al [6,45]. 

To compare the idea of this manuscript with the other reports in the literature [22,24,28], this study especially focused on detection of the surface-confined interaction between 2,4-D and DNA based on the guanine signal by using PGEs and evaluate the DNA damage occurred by 2,4-D under different experimental conditions. Azadmehr and Zarei [24] reported an indicator based electrochemical biosensor whose fabrication required many labor-intensive experimental steps, different chemical agents, long time (at least 2 hour). 

In the study reported by Ahmadi and Bakhshandeh [28], mercury drop electrode was used as the biosensor surface and electrodeposition of 2,4-D was performed. Then, the interaction was done by adding DNA. Although the interaction mechanism of 2,4-D and DNA was compherensively investigated by using not only electrochemical but also spectroscopic techniques, the oxidation signal of guanine was not measured and evaluated by means of interaction process, and the recognition system reported in that study was not labor and enviromental friendly and was not miniaturizable compared to the electrochemical biosensor developed in this study. Moreover, pencil graphite electrodes (PGEs) provide disposable, easy-to-use, and robust surfaces to immobilize biomolecules compared to the other electrodes used for the purpose of detection of 2,4-D [15-24]. The preparation of PGEs requires only 30 s, the immobilization of DNA could be completed in 15 min and the interaction was accomplished in 5 min. 

Single cell gel electrophoresis assay (SGCE) method has been widely used for evaluation of the genotoxicity of the 2,4-D in in vitro [6,58,59]. In this method, cell culture should be prepared. The incubation time of the cells with 2,4-D should be quite longer and this method requires the preparation of agarose gel and employment of electrophoresis. The single-use electrochemical biosensor platform eliminates all disadvantages of the SGCE such as being longer and to require labor-intensive experimental steps. Moreover, the biosensor platform is adaptable to chip technology. 

## 4. Conclusion 

The Detection of the interaction between DNA and 2,4-D was investigated by using a disposable biosensing platform in the scope of this study. After the optimization of the experimental parameters, the interaction was investigated at different concentration of 2,4-D. The interaction of 2,4-D and DNA at the surface of PGE was characterized by using microscopic and electrochemical techniques, consistent results were obtained. The interaction of DNA and GLY was also studied and the highest decrease at the guanine signal could be monitored in the presence of 2,4-D. Taking a step further, the interaction was investigated in the presence of the herbicide sample. 

This report will lead to fabrication of miniaturized systems that allow the monitoring of the interaction of DNA and different herbicides/pesticides in the future. Furthermore, this study will bring a new perspective into environmental safety researches.
